# The *SDHD*:p.H102R Variant Is Frequent in Russian Patients with Head and Neck Paragangliomas and Associated with Loss of 11p15.5 Region and Hypermethylation of H19-DMR

**DOI:** 10.3390/ijms24010628

**Published:** 2022-12-30

**Authors:** Anastasiya Snezhkina, Maria Fedorova, Anastasiya Kobelyatskaya, Daria Markova, Margarita Lantsova, Anna Ikonnikova, Marina Emelyanova, Dmitry Kalinin, Elena Pudova, Nataliya Melnikova, Alexey Dmitriev, George Krasnov, Vladislav Pavlov, Anna Kudryavtseva

**Affiliations:** 1 Engelhardt Institute of Molecular Biology, Russian Academy of Sciences, Moscow 119991, Russia; 2 Vishnevsky Institute of Surgery, Ministry of Health of the Russian Federation, Moscow 117997, Russia

**Keywords:** head and neck paraganglioma, mutation frequency, SDHD, LOH, H19-DMR, KvDMR, 11p15

## Abstract

Head and neck paragangliomas (HNPGLs) are rare neuroendocrine neoplasms derived from the parasympathetic paraganglia of the head and neck. At least 30% of HNPGLs are linked to germline mutations, predominantly in *SDHx* genes. In this study, we analyzed an extended cohort of Russian patients with HNPGLs using whole-exome sequencing and found a highly frequent missense variant p.H102R in the *SDHD* gene. We determined this variant in 34% of the *SDHD* mutation carriers. This variant was associated with somatic loss of the gene wild-type allele. Data from the B allele frequency method and microsatellite and microdeletion analysis indicated evident LOH at the 11p15.5 region and potential loss of the whole of chromosome 11. We found hypermethylation of H19-DMR in all tumors, whereas differential methylation of KvDMR was mostly retained. These findings do not support the paternal transmission of *SDHD*:p.H102R but are in agreement with the Hensen model. Using targeted sequencing, we also studied the variant frequency in a control cohort; we found *SDHD*:p.H102R in 1.9% of cases, allowing us to classify this variant as pathogenic. The immunohistochemistry of SDHB showed that the *SDHD*:p.H102R mutation, even in combination with wild-type allele loss, does not always lead to SDH deficiency. The obtained results demonstrate the frequent variant associated with HNPGLs in a Russian population and support its pathogenicity. Our findings help with understanding the mechanism of tumorigenesis and are also important for the development of cost-effective genetic screening programs.

## 1. Introduction

Paragangliomas and pheochromocytomas (PPGLs) are rare neuroendocrine tumors originating from the paraganglia of the sympathetic and parasympathetic nervous systems [1]. Parasympathetic paragangliomas (PGLs) predominantly develop in the head and neck region and are less frequently located in the thorax and pelvis. Sympathetic PGLs primarily arise from the adrenal medulla and are called pheochromocytomas (PHEOs) but may also have extra-adrenal localization.

Head and neck paragangliomas (HNPGLs) account for approximately 0.03% of all tumors and are designated depending on their anatomic site of origin: bifurcation of the carotid arteries (carotid paraganglioma (CPGL)), the middle ear (middle ear paraganglioma (MEPGL)), along the vagus nerve (vagal paraganglioma (VPGL)), and in the larynx (laryngeal paraganglioma (LPGL)) [2]. HNPGLs occur as either single or multifocal tumors and have a predominance in women [1]. Metastasizing HNPGLs are diagnosed in 2–19% of cases depending on tumor localization, but all HNPGLs have the potential to metastasize [3]. HNPGLs are characterized by high heritability, mostly associated with mutations in *SDHx* (*SDHA*, *SDHB*, *SDHC*, and *SDHD*) genes, which encode for succinate dehydrogenase (SDH) subunits, and in the *SDHAF2* gene. HNPGLs with germline mutations in these genes can occur as paraganglioma syndrome type 1–5, which has a familial predisposition to tumorigenesis [4]. PGL syndromes develop as autosomal dominant diseases with highly variable penetrance. A combination of numerous internal and external factors can affect the penetrance of *SDHx*-related tumors, including the imprinted transmission of *SDHD* and *SDHAF2* mutations, which are almost always characterized by paternal inheritance [5]. However, the molecular genetic mechanisms of incomplete penetrance for *SDHx* mutations are still not well understood.

In general, at least 20 susceptibility genes for PPGLs are known, most of which are associated with hereditary syndromes [6]. Thus, genetic testing is recommended for all patients with PPGLs on a multigene panel (*SDHx*, *SDHAF2*, *RET*, *FH*, *VHL*, *NF1*, *MEN1*, *TMEM127*, and *MAX*) [7,8]. Moreover, targeting genetic examination at *SDHB* or *SDHD*, at minimum, should be suggested for patients with HNPGLs. Pathogenic *SDHB* and *SDHD* mutations increase the risk of metastasis and the development of multifocal tumors, respectively [3]. Data on the presence of a hereditary mutation predisposing an individual to tumor development and/or predicting its aggressive potential are crucial for early diagnosis of tumors in asymptomatic mutation carriers, active surveillance of patients with high metastatic or multifocal potential, as well as determining an effective treatment strategy.

We recently revealed that, among all PPGL susceptibility genes, *SDHD* is the most frequently mutated in Russian patients with HNPGLs [9]. In the current study, we extended the number of genetically tested Russian patients and showed the high prevalence of the *SDHD*:p.H102R missense variant in the cohort. We also analyzed the frequency of this variant in a cohort of healthy individuals. We examined the loss of various regions on chromosome 11, including the *SDHD* gene locus and the 11p15.5 region, which is thought to contain the imprinted second tumor suppressor gene (TSG) involved in tumor formation, explaining the phenomenon of paternal transmission of *SDHD*-mutated PPGLs. In addition, we estimated the effect of *SDHD*:p.H102R on the destabilization of the SDH complex. This study is particularly relevant in the context of genetic screening and counseling, as well as understanding the mechanisms through which *SDHD*-linked tumorigenesis occurs.

## 2. Results

### 2.1. Clinical Characteristics and Identification of the SDHD:p.H102R Variant

A total of 144 tumors from 134 patients with HNPGLs and 373 normal tissues from healthy individuals were included in the present study. The age of the patients in the case cohort ranged from 16 to 84 years old (mean age 48.3 years; median age 49 years); the ratio of women to men was ~3:1. The clinicopathologic characteristics of the case cohort are presented in Table 1. The control cohort included healthy individuals without any clinical signs of tumors who were aged from 25 to 94 years old (mean age 70.2 years; median age 71 years); the ratio of women to men was ~2:1. The mean and median age of the control cohort corresponded to those when age-related penetrance of the *SDHD*-associated PPGLs was reported to reach 87–100% [7,10,11].

The results of whole-exome sequencing of the expanded case cohort revealed the following number of *SDHx* variants in patients with HNPGLs: 2 *SDHA*, 15 *SDHB*, 5 *SDHC*, and 35 *SDHD*. The prevalence (number of subjects having the variant to the total number of subjects with HNPGLs) of *SDHx* variants in Russian patients with HNPGLs was 42.5% (57/134), which is consistent with our previous result [9].

The most frequent variant was a missense variant in the *SDHD* gene, NM_003002: c.A305G, p.H102R (chr11:111959726, rs104894302), which we detected in 14 tumor tissues obtained from 12 patients (1 patient had multifocal HNPGLs manifesting as two bilateral carotid PGLs and a vagal PGL (001tc1, 001tc2, and 001tv)) [12] (Table 2). We found this variant in 34% (12/35) of patients with *SDHD* mutations and 21% (12/57) of all *SDHx*-mutated individuals in the studied case cohort. The *SDHD*:p.H102R variant accounted for approximately 9% of all HNPGLs. We determined the variant as germline in almost all cases (10 of 11 patients tested); only in 1 patient (022tc) did we confirm the somatic status of the variant. The frequency of *SDHD*:p.H102R was a little higher in vagal paragangliomas than in carotid paragangliomas (~12% vs. 9%).

One patient with *SDHD*:p.H102R (100tc) had an additional germline variant in the *NF1* gene, characterized by conflicting interpretations of pathogenicity. According to VarSome search engine and the American College of Medical Genetics and Genomics and the Association for Molecular Pathology (ACMG-AMP), this variant was interpreted as a variant of uncertain significance. Hereditary PPGLs are usually connected to a single pathogenic germline mutation in one of the susceptibility genes; thus, the identified NF1 variant was likely benign in this patient. Patients harboring the *SDHD*:p.H102R variant were characterized by a mean age of onset of 50.4 years (median age 50.2 years) and were predominantly women patients (11/12).

Among the tested control cohort, we determined the *SDHD*:p.H102R heterozygous variant in six healthy individuals, indicating the higher frequency (1.6%, 6/373) of this variant in this Russian population compared with those in the European population (0.0009%) reported by The Genome Aggregation Database (gnomAD). The age of the identified healthy *SDHD*:p.H102R mutation carriers ranged from 66 to 80 years (mean age 72.8 years; median age 72.5 years) with a ratio of women to men of 1:1.

### 2.2. Copy Number Variations (CNVs) and Loss of Heterozygosity (LOH) in Chromosome 11

**Figure 1:**
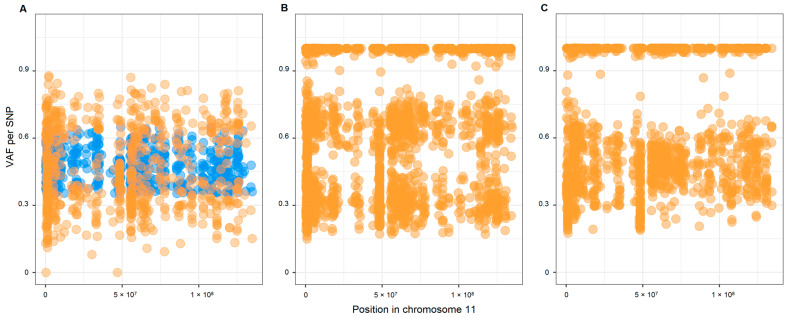
VAF plots of chromosome 11 in HNPGL patients with *SDHD*:p.H102R variant. (**A**) Potential loss of chromosome 11 identified in patient 120tv (BAF analysis for tumor vs. matched normal tissue); (**B**) potential loss of chromosome 11 identified in patient 56tc (BAF analysis for tumor only); (**C**) two copies of chromosome 11 identified in patient 135tc (BAF analysis for tumor only). Blue dots, heterozygous SNPs in normal tissue; orange dots, heterozygous SNPs in tumor.

Based on the B allele frequency (BAF) method, we analyzed the CNV in chromosome 11. This analysis was available for 12 tumors, and all except 1 (135tc) showed a potential loss of chromosome 11. An example of the potential loss of chromosome 11 and, conversely, the presence of two copies of chromosome 11 in the studied patients are shown in Figure 1.

To verify the deletion of regions on chromosome 11, we analyzed the microsatellite markers and potential microdeletions. We chose the microsatellite markers from telomeric regions (11p15.5 and 11q25), centromeric region (11q12.1), loci close to the *SDHD* gene (11q22.1, 11q22.3, and 11q23.1), as well as two random regions on the p arm (11p13) and q arm (11q14.1). We analyzed eleven patients at every marker. The frequency of LOH for 11 of the 12 studied markers ranged from about 70% to 100%. One locus (D11S4088) located proximal to 11p15.5 was characterized by the lowest LOH frequency of 45%; the most distal marker (D11S1984) showed a 92% LOH frequency.

**Figure 2:**
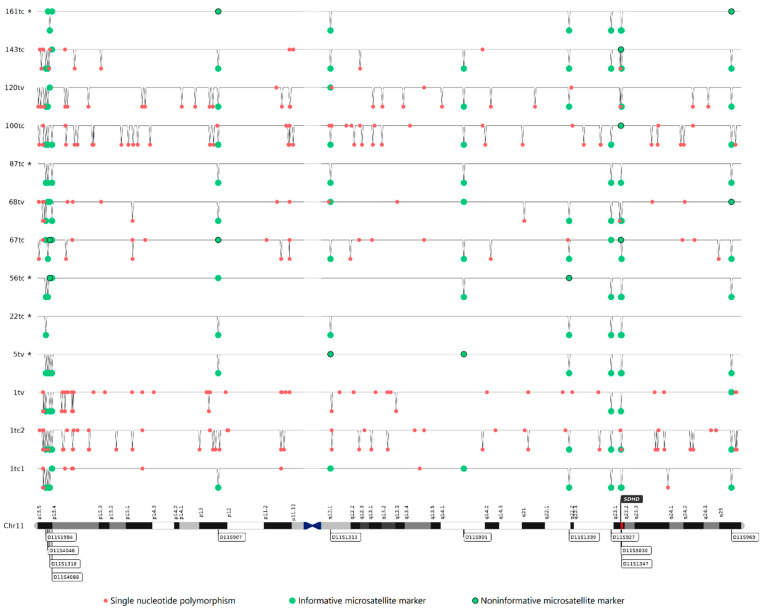
Visualization in chromosome 11 abnormalities in patients with *SDHD*:p.H102R variant. Red and green dots mark SNPs and microsatellites, respectively. Red and green dots on line indicate retention of allele and those under the line show acquisition of homozygosity and loss of heterozygosity of SNPs and microsatellites, respectively. * Patients analyzed for microsatellite markers only.

We studied microdeletions in eight patients based on exome sequencing data for paired tumor–normal tissues using the variant allele frequency (VAF) imbalance of germline heterozygous SNPs. We mapped individual SNPs, passing the filter, on chromosome 11 as either one of two states: retention of allele (heterozygosity) or acquisition of homozygosity, where the latter indicates potential microdeletion (Figure 2). Considering intratumor heterogeneity and the admixture of normal cells, we considered acquisition of homozygosity if it occurred in not less than 30% of the cells. We considered allele retention when it was retained in at least in 98% of the cells. Note that in this method, frequent microdeletions reflected only regions on chromosome 11 that were deleted in more tumor cells than in other regions. We observed the highest frequency of microdeletions in 11q23.1 (mean value 88% (50–100)), 11q21 (70% (0–100)), 11p15.5 (65% (22–100)), 11p13 (65% (17–100)), and 11q15.1 (55% (0–100)). In general, the numerous microdeletions and LOH at most microsatellite markers supported the potential loss of chromosome 11 revealed by BAF analysis in all studied patients.

### 2.3. Maternal Allele Specific Loss of the 11p15.5 Region

We performed target DNA methylation sequencing of the differentially methylated region (DMR) upstream of the *H19* gene (H19-DMR) and the KvDMR region, which are located at the 11p15.5 imprinted gene cluster. KvDMR is methylated on the maternal allele and expressed from the paternally inherited chromosome 11; H19-DMR is methylated on the paternal allele and characterized by expression from the maternally inherited chromosome 11. Thus, in normal and tumor tissues with two chromosomes, the methylation rate (MR) of both regions should be close to 50%. In the case of maternal allele loss, the methylation rate for H19-DMR and KvDMR is expected to tend to be 100% and 0%, respectively.

**Figure 3:**
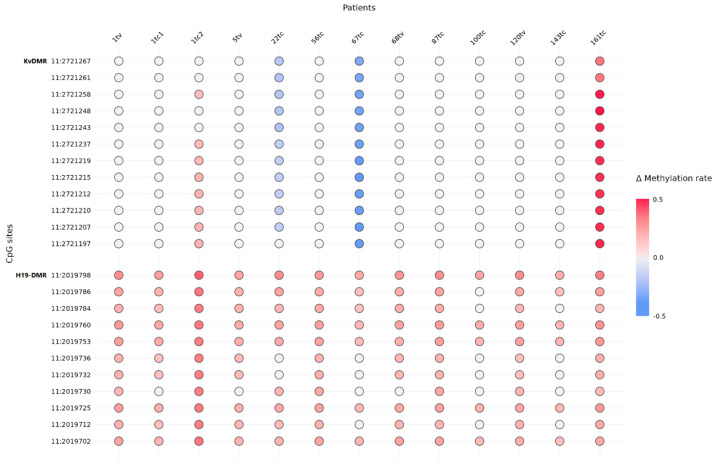
Delta methylation rate of CpG sites within H19-DMR and KvDMR between tumor and normal tissues of *SDHD*:p.H102R-mutation carriers. Color saturation corresponds to delta methylation rate. Genomic coordinates of CpG sites refer to GRCh37/hg19 assembly.

After bioinformatics analysis of sequencing data and filtering, we obtained 11 and 12 representative CpG sites at H19-DMR and KvDMR, respectively (Figure 3). The mean MR value in normal tissues was 56% and 45% for H19-DMR and KvDMR, respectively. We considered the change in the beta value of MR between the tumor and normal ≥ 15% (*p* ≤ 0.05) as hyper- or hypomethylation. In the tested cohort, we revealed hypermethylation of H19-DMR in all tumors, whereas the MR of CpG cites at KvDMR regions was predominantly unchanged compared with that of normal tissue. Only two patients (22tc and 67tc) were simultaneously characterized by hypermethylation of H19-DMR and hypomethylation of KvDMR. Another patient (161tc) and one of three tumors from the patient with multifocal HNPGLs (1tc2) displayed hypermethylation of H19-DMR and hypermethylation of KvDMR.

### 2.4. SDH Complex Deficiency

We subjected the FFPE tumor tissues obtained from patients harboring the *SDHD*:pH102R variant to IHC analysis of the expression pattern of the SDHB subunit (Table 2). We found negative or weak diffuse SDHB expression in 57% (8/14) of tumors, indicating SDH complex deficiency. In one patient (143tc), we observed a heterogeneous SDHB expression pattern presented as regions with positive and negative SDHB IHC staining, which can be caused by different cell population within the tumor.

## 3. Discussion

In this study, we noted a highly frequent missense variant p.H102R in the *SDHD* gene associated with HNPGLs in Russian patients. We determined the variant in about 9% of HNPGLs, which accounted for approximately one-quarter of the identified *SDHx* mutations. The *SDHD*:p.H102R mutation was previously submitted to ClinVar as a germline variant related to PPGLs, gastric stromal sarcoma, Cowden syndrome 3, and hereditary cancer-predisposing syndrome. This variant was classified by the autosomal dominant and X-linked criteria (v.2-20-17) as ‘likely pathogenic’, when the Invitae variant classification Sherloc has interpreted it as ‘uncertain significance’ [13]. We initially classified *SDHD*:p.H102R as a ‘likely pathogenic’ variant using the following available ACMG-AMP criteria: PM1, PM2, PM5, PP3, and PP5 [14]. To verify criterion PS4 (strong evidence of variant pathogenicity), we estimated the frequency of the variant in the Russian population of healthy individuals (race-matched control data). The odds ratio (OR) obtained from the case–control study was 6.02 (CI = 2.21–16.37). According to ACMG-AMP, the PS4 criterion may be applied for variants passing the OR threshold of >5. Thus, the addition of the PS4 criterion allows classifying *SDHD*:p.H102R as ‘pathogenic’. Notably, the VarSome tool also classified this variant as ‘pathogenic’.

Germline *SDHD*:p.H102R variant was also detected in an earlier independent study of 91 Russian patients with HNPGLs reported by Shulskaya et al. [15]. Three patients (all with multifocal tumors) harbored this mutation. In our cohort, we found *SDHD*:p.H102R in only one case of multifocal tumor that was previously described [12]. The prevalence of the variant in a previous study was approximately 3% and was equal to the frequency of synonymous substitution p.S68S, which was also determined in the *SDHD* gene and predicted as potentially influencing splicing. In our cohort, the frequency of *SDHD*:p.H102R was three times higher; this can be explained by the predominance of MEPGLs in the study of Shulskaya et al., whereas tumors of this localization were not included in our case cohort. Importantly, *SDHD*:p.H102R variant was also found in a case of malignant CPGL with multiple metastases [16]. Thus, the literature data and our results indicate that patients carrying this variant are predisposed to multifocal and malignant HNPGLs and, therefore, need long-term follow-up.

The high prevalence of the *SDHD*:p.H102R variant in the Russian population but an extreme rarity in other populations suggests its potential founder effect. In the literature, several founder mutations are reported to be responsible for PPGLs. Two founder mutations, p.D92Y and p.L139P were revealed in familial and isolated cases related to a Dutch population [17]. Among the 205 *SDHD* mutation carriers, the p.D92Y and p.L139P variants were detected in 81% and 13% of cases, respectively, all of which predominantly caused HNPGLs [18].

The *SDHD*:p.H102R variant was found in 34% of *SDHD* mutation carriers with HNPGLs from Russia, which is three times higher than that of the second most dominant mutation (p.L139P) in the Netherlands. Russian patients were characterized by a higher frequency of *SDHD* mutations, whereas the Dutch cohorts showed a predominant frequency of *SDHB* variants [9,19]. One more founder mutation in the *SDHD* gene, p.Q109X, was found in Italy [20], and a potential founder large *SDHD* deletion was identified in Austrian families [21]. Thus, *SDHD* founder mutations predisposing to PPGLs seem to be specifically distributed in several populations due to historical reasons and geographical differences.

Germline *SDHD* mutations cause familial paraganglioma syndrome type 1 (PGL1) and can be associated with apparently sporadic tumors [22]. *SDHD*-linked PPGLs are transmitted in an autosomal dominant fashion with a parent-of-origin effect when disease occurs if the mutation is inherited from the father [23]. However, no evidence exists of genetic imprinting for the *SDHD* locus, and the gene has biallelic expression in several human tissues [24]. Additionally, paternally transmitted mutations with somatic loss of the normal maternal *SDHD* region is shown in affected individuals, suggesting *SDHD* as a tumor suppressor gene that requires both alleles to be inactivated following Knudson’s two-hit model [24,25]. Of note, another study showed the loss of the entire copy of maternal chromosome 11 in patients with PPGLs [26]. Hensen et al. hypothesized that a second imprinted TSG gene located at the 11p15 region may be involved in tumorigenesis [26]. The maternal transmission of this gene explains the need for the loss of the entire maternal chromosome 11 for tumor formation and the low frequency of maternal inheritance of the *SDHD* mutations. Thus, only several cases with the inheritance of disease from the mother have been reported, and all confirmed that the development of *SDHD*-mutated tumors is consistent with the Hensen model [27,28,29,30].

**Figure 4:**
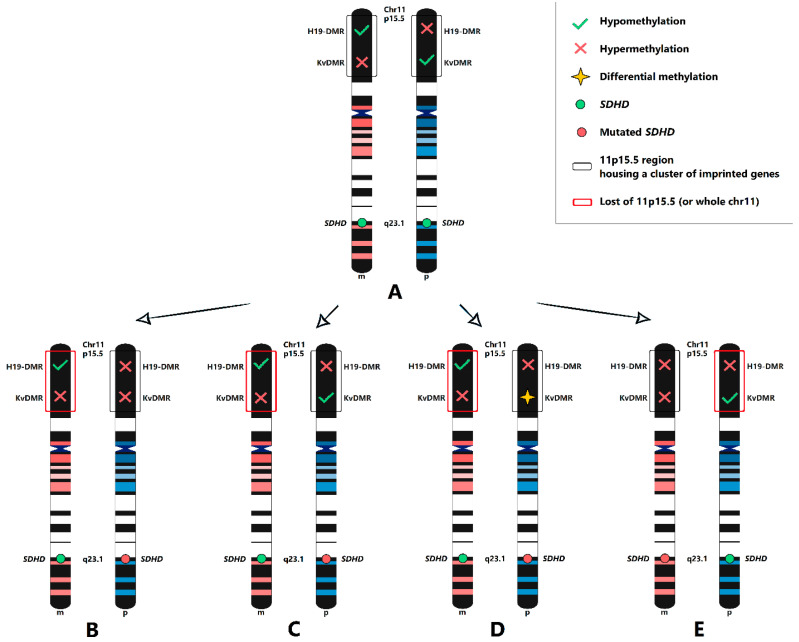
Scheme of putative genetic changes within 11p15.5 locus associated with *SDHD*:p.H102R variant. (**A**) Nonmutated maternal (m) and paternal (p) chromosome 11 with normal methylation of H19-DMR and KvDMR. The 11p15.5 locus is lost on maternal chromosome, whereas mutated paternal chromosome carries hypermethylated H19-DMR and hypermethylated KvDMR (**B**), hypermethylated H19-DMR and hypomethylated KvDMR (**C**), and hypermethylated H19-DMR and differently methylated KvDMR (**D**). (**E**) Mutated maternal chromosome carries hypermethylated H19-DMR and hypermethylated KvDMR, while paternal chromosome lacks them in 11p15.5 region.

In this study, we examined the loss of putative disease-associated regions and the potential loss of the whole of chromosome 11 in patients with the *SDHD*:p.H102R mutation. We revealed evident somatic loss of heterozygosity in regions close to the *SDHD* gene; this finding indicated that *SDHD* acts as a TSG and is tumorigenic in accordance with Knudson’s hypothesis. The patient with a somatic mutation also carried LOH at the *SDHD* locus; therefore, nonhereditary HNPGLs may also be driven by somatic allele loss of the susceptibility gene (in our case, *SDHD*). We also determined loss of heterozygosity at the 11p15.5 locus, which includes a large imprinted gene cluster hypothetically containing a second TSG, the inactivation of which can contribute to tumor development. The results obtained from microsatellite markers and microdeletion analysis, together with data from BAF analysis, indicated the potential loss of chromosome 11, which provides evidence to the Hensen model. However, in most cases, we did not find the expected change in the methylation rate of H19-DMR and KvDMR imprinted regions, which can fully confirm the loss of the maternal chromosome 11. All tested tumors were characterized by hypermethylation in the H19-DMR region when we observed a differential methylation rate for KvDMR. On one hand, hypermethylation of H19-DMR is consistent with evident LOH at the 11p15.5 region on the maternal chromosome 11 (Figure 4B–D). On the other hand, if the maternal 11p15.5 region (or whole chromosome 11) is lost, KvDMR should undergo either hypermethylation or differential methylation on the paternal chromosome (Figure 4C,D). Therefore, two cases similar to ours were reported in *SDHB*-mutated PPGLs having hypermethylation of H19-DMR and normal methylation of KvDMR with LOH at the 11p15 locus [31]. One more possible variant explaining the hypermethylation of both H19-DMR and KvDMR found in the two patients studied is the maternal transmission of the *SDHD*:p.H102R mutation (Figure 4E). In this case, LOH at 11p15.5 can occur on the paternal chromosome, while the imprinted hypermethylated KvDMR and somatically hypermethylated H19-DMR are located at maternal chromosome 11. This is consistent with previous findings showing hypermethylation of a CpG site within H19-DMR in a rare case of maternally transmitted *SDHD*-mutated HNPGL [27].

We also studied the effect of the *SDHD*:p.H102R variant on the SDH complex. Inactivating *SDHx* mutations typically affects the protein function, which can lead to the loss of the corresponding SDH subunit, resulting in decreased stability of the SDH complex [32]. The SDHB subunit releases from the unstable SDH complex into the cytoplasm and degrades there, as can be detected by negative or weak diffuse SDHB immunostaining [33]. A little more than half of tumors with the *SDHD*:p.H102R variant showed negative or weak diffuse staining of the SDHB subunits regardless of the sex of the patient or tumor localization. In almost all cases with positive SDHB expression (excepting one, 135tc), we found a loss of the wild-type allele of the *SDHD* gene. Moreover, one tumor (143tc) showed intratumoral immunohistochemistry heterogeneity and was characterized by both positive and negative SDHB staining. Possibly, the p.H102R mutation in the *SDHD* gene and subsequent LOH at the gene locus cannot lead to its complete inactivation. The affected SDHD subunit may improperly act in the SDH complex without a visible deficiency but may regardless leading to tumorigenesis. SDH complex deficiency can arise later from the accumulation of additional changes caused by insufficient SDH activity over time.

## 4. Materials and Methods

### 4.1. Human Tissues

We studied two human sample sets: (1) 144 tumor tissues from 134 Russian patients with HNPGLs (case cohort) that we expanded to compare with our previous findings, and (2) 373 blood tissues from healthy Russian adults (control cohort). We obtained written informed consent from all individuals. The study was approved by the Ethics Committee of the Vishnevsky Institute of Surgery and performed according to the Declaration of Helsinki.

### 4.2. DNA Isolation

We isolated DNA from FFPE tissues (tumors or lymph nodes) using a High Pure FFPET DNA Isolation Kit (Roche, Basel, Switzerland) according to the manufacturer’s protocol. We extracted DNA from the blood samples with a MagNA Pure Compact Nucleic Acid Isolation Kit I (Roche) on a MagNA Pure Compact Instrument (Roche). We quantified DNA with a Qubit 4.0 fluorometer (Thermo Fisher Scientific, Waltham, MA, USA).

### 4.3. Whole-Exome Sequencing of Case Cohort

We subjected the expanded case cohort to whole-exome sequencing to estimate the mutational status of the *SDHx* genes. We prepared exome libraries from tumors and matched normal tissues (lymph node or blood samples) using a TruSeq Exome Kit (Illumina, San Diego, CA, USA) according to the manufacturer’s protocol, which we sequenced on an Illumina NextSeq 500 System using an Illumina NextSeq 500/550 High Output Kit v2.5 (150 cycles) in paired-end mode, 2 × 76 bp. The average coverage for each sample was at least 300×.

We performed bioinformatics analysis of exome data as previously described [34]. We used GATK HaplotypeCaller 4.2.4.0 for variant calling [35]. We annotated the variants using ANNOVAR [36] supplemented with additional data, including population frequency, conservation score, prediction score of pathogenicity, and clinical significance. We also analyzed the variant pathogenicity using the VarSome The Human Genomics Community [37], and we drew conclusions based on criteria of the American College of Medical Genetics and Genomics and the Association for Molecular Pathology [14].

### 4.4. Genetic Testing of Control Cohort

We performed genetic testing of the presence of the target variant in the control cohort using amplicon sequencing. We prepared the amplicon library with two-stage PCR. We designed the first-stage PCR primers, carrying the 5′ sequences complementary to the adapter sequences, for amplification of the target region in the *SDHD* gene containing the p.H102R variant: forward, TCGTCGGCAGCGTCAGATGTGTATAAGAGACAGTTTAGGGCATTTCAATCAACTTCTC; reverse, GTCTCGTGGGCTCGGAGATGTGTATAAGAGACAGGGACTAGCGAGAGGGTTGTC. We used the second-stage PCR primers for dual-index barcoding of the target amplicon.

We performed all PCR reactions with a Tersus Plus PCR kit (Evrogen, Moscow, Russia). We ran the amplification program of the first stage PCR on a T100 Thermal Cycler (Bio-Rad, Hercules, CA, USA) with the following scheme: 95 °C for 3 min, 30 cycles of 95 °C for 30 s, 56 °C for 30 s, and 72 °C for 30 s, then 72 °C for 5 min, and holding at 4 °C. We ran the obtained amplicons on 2% agarose gel electrophoresis to verify the size. Then, we purified the amplicons using KAPA Pure Beads (Roche) according to the manufacturer’s protocol. We applied a second amplification round for double-indexing of the samples. The amplification program was as follows: 95 °C for 3 min, 8 cycles of 95 °C for 30 s, 55 °C for 30 s, and 72 °C for 30 s, then 72 °C for 5 min, and holding at 4 °C. After a second clean up, we measured the concentration of the libraries with a Qubit 4.0 fluorimeter. We mixed the purified amplicons in equimolar proportions, which we validated with an Agilent 2100 Bioanalyzer (Agilent Technologies, Santa Clara, CA, USA). The size of finale-pooled library was ~360 bp. We sequenced the library on an Illumina MiSeq System using an Illumina MiSeq Reagent Nano Kit v2 (300-cycles) in paired-end mode, 2 × 151 bp. We obtained no less than 2000 reads for each sample.

We performed sample demultiplexing and generation of fastq files using MiSeq Reporter Software (Illumina). We assessed the quality of the reads with FastqQC [38]. Then, we removed the remaining adapter and primer sequences using cutadapt [39]; finally, we trimmed the sequences with Trimmomatic [40]. We then aligned the reads against the GRCh37/hg19 human reference genome using BWA-MEM [41]. We did not perform the procedure for marking duplicates. We performed variant calling using freeBayes [42] and GATK HaplotypeCaller 4.2.4.0 [35] (with disabled duplicated reads filter, disabled strand bias, and odds ratio filtration). The average amplicon coverage was 4800×.

### 4.5. Copy Number Variation Analysis

We performed the analysis of CNV on chromosome 11, where the studied gene, *SDHD*, is located (11q23.1), using the beta allele frequency method [34]. Briefly, we filtered heterozygous SNPs in normal tissues with the following parameters: (1) read coverage depth > 25; (2) variant allele frequency = 0.35–0.65; and (3) availability of any dbSNP annotation. We plotted the VAF values for the SNPs that met the requirements. We considered the difference between VAF values (tumor–normal tissues) statistically significant when it was >0.15 and Fisher’s exact test *p* < 0.05. For tumors without paired norms, we only plotted the VAF values and looked up whether the VAF distribution was split. For large CNVs (whole chromosome or an arm), the VAF distribution (more than 0.65 and less than 0.35) was split, and such cases were obvious.

In addition, we analyzed the microdeletions on chromosome 11. The differences in allele frequencies of germline heterozygous SNPs in their tumor and paired normal tissues that are close to 0.5 indicate the acquisition of homozygosity in the tumor sample and potential microdeletions. On the contrary, retention of VAF values in tumor and normal tissues is considered a conservation of the wild-type region.

### 4.6. Loss of Heterozygosity Analysis

To determine the LOH at the *SDHD* locus (11q23.1), we analyzed 12 highly polymorphic (heterozygosity > 0.7) dinucleotide microsatellite markers for the following regions on chromosome 11: 11p15.5 (D11S1984, D11S4046, D11S4088, D11S1318), 11p13 (D11S907), 11q12.1 (D11S1313), 11q14.1 (D11S901), 11q22.1 (D11S1339), 11q22.3 (D11S927), 11q23.1 (D11S5030, D11S1347), and 11q25 (D11S969). We analyzed all markers in tumors and matched normal tissues (blood or lymph node). We synthesized primers as paired primer sets of labeled forward primer with fluorescent tag on the 5′ end and unlabeled reverse primer (Syntol, Moscow, Russia). The sequences of primers are listed in Table S1.

We diluted the DNA from the paired samples to equal concentrations. We used 50–100 ng of this DNA per amplification reaction. The total volume of 25 μL included 2.5 μL of 10× Tersus buffer, 0.5 μL of 50× Tersus polymerase, 0.5 μL of 10 mM dNTP (all from Tersus Plus PCR kit, Evrogene, Russia), 0.5 μL of 10 μM labeled forward primer and 2 μL of 10 μM unlabeled reverse primer (1:4 ratio), 3 μL of template DNA, and 16 μL of PCR-grade water (Thermo Fisher Scientific, USA). We performed PCR on a T100 Thermal Cycler (Bio-Rad, USA) using the following protocol: predenaturation at 95 °C for 5 min; 30 cycles of denaturation at 94 °C for 30 s, annealing at 48–55 °C (depending on primer pair) for 30 s, and extension at 72 °C for 30 s, followed by a final extension at 72 °C for 7 min, and holding at 4 °C. We validated the PCR products using 2% agarose gel, which we then cleaned up using KAPA Pure Beads (Roche) according to the instructions. We performed fragment analysis of amplicons with a NANOPHORE-05 (Syntol). We used GeneMarker software (SoftGenetics, State College, PA, USA) for the visualization and LOH analysis.

### 4.7. Immunohistochemistry (IHC)

We investigated the FFPE tumor samples with IHC staining of the SDHB subunit as previously described [33]. We performed immunoreactions using mouse monoclonal antibody 21A11AE7 (Abcam, Boston, MA, USA), followed by Mayer’s hematoxylin staining. We scanned the slides with a PANNORAMIC 250 Flash III scanner (3DHISTECH, Hungary). We assessed the granular cytoplasmic SDHB staining of the tumor cells as positive if the internal positive control (endothelial cells) was stained in the same way. We scored negative staining when SDHB expression was completely absent in tumor cells together with positive staining of the endothelial cells. We recorded the expression pattern as weak diffuse when slides were stained as a cytoplasmic blush without definite granularity, but with strong granular staining of the internal positive control.

### 4.8. DNA Methylation Analysis

We subjected the genomic DNA (200 ng) isolated from paired tissues (and normal tissue derived from the same patient) to enzymatic conversion to detect modified cytosines using a NEBNext Enzymatic Methyl-seq Conversion Module (NEBNext, Ipswich, MA, USA) according to the manufacturer’s directions. We used the complete absence of the product after PCR amplification of converted DNA with regular nonmodified DNA-specific primers as a control of the successful DNA conversion. We amplified the converted DNA by PCR using two pairs of primers overlapping the 11 and 15 CpG sites within the H19-DMR and KvDMR regions on chromosome 11, respectively, designed for the first PCR step of sequencing library preparation (Table S2). We used the following program w for the first PCR amplification: 95 °C for 3 min, 40 cycles of 95 °C for 30 s, 54 °C for 30 s, and 72 °C for 30 s, then 72 °C for 5 min, and holding at 4 °C. We visualized the obtained amplicons on 2% agarose gel to confirm their expected length. Then, we processed the amplified DNA fragments for the following steps of targeted library preparation, as well as for quantity and quality control as described above. We measured the size of the final libraries with capillary electrophoresis, which was about 300 bp.

We performed the paired-end sequencing of the libraries (250 × 2 bp) using a MiSeq System (Illumina). The sequence depth was approximately 1000× for each targeted region in each sample. The bioinformatics analysis included the following steps: (1) quality control, (2) adapter and low-quality read trimming, (3) alignment on in silico converted reference sequence and cytosine methylation calling using Bismark [43], (4) calculation of the methylation level for each individual CpG site (ratio of number of reads with unconverted cytosines mapped at the individual CpG site to the total number of reads covering this position), and (5) evaluating conversion efficiency as previously described [44]. DNA conversion efficiency was at least 99% for each sample.

## 5. Conclusions

In this study, we uncovered the highly frequent pathogenic *SDHD*:p.H102R variant associated with HNPGLs in the Russian population. This finding should be considered in the development of effective screening programs in asymptomatic people and/or affected individuals. Moreover, we showed that tumorigenesis is triggered by the loss of the wild-type allele in *SDHD*:p.H102R-mutated hereditary and nonhereditary HNPGLs, and is associated with LOH at the 11p15.5 locus (or potential loss of the whole of chromosome 11) and hypermethylation of H19-DMR. Additionally, we think that the *SDHD*:p.H102R mutation, even in combination with LOH at the gene locus, leads to incomplete protein inactivation, but this is enough for tumors to develop.

## Figures and Tables

**Table 1:** Clinicopathologic characteristics of case cohort.

Characteristics		Data
Age, years	Min–max	16–84
	Mean/median	48.3/49
Sex, n (%)	Female	101 (75%)
	Male	33 (25%)
Tumor localization, n (%)	Carotid body *	103 (77%)
	Along the vagus nerve *	33 (25%)
	N/A	3 (2%)
Tumor type, n (%)	Recurrent	1 (0.7%)
	Metastatic	2 (1.3%)
	Multifocal	10 (7.5%)
Total, n	Patients	134
	Tumors	144
*SDHx* variant, n (%)	*SDHA*	2
	*SDHB*	15
	*SDHC*	5
	*SDHD*	35

* Patients with multifocal paragangliomas localized at carotid body and near vagus nerve were counted in both groups. N/A, data unavailable.

**Table 2:** The *SDHD*:p.H102R variant carriers of the case cohort.

Patient ID	Sex	Age, Year	Tumor Localization	*SDHD*:p.H102R Mutation Status	Variant in Other PPGL Susceptibility Genes	BAF-Analysis Result	SDHB IHC Staining
1tc1 1tc2 1tv	F	58	Multifocal (bilateral carotid body tumor and unilateral vagal tumor)	Germline	-	Chr 11 − (all three tumors)	Weak diffuse (all three tumors)
5tv	F	61	Vagal	Germline	-	Chr 11 −	Negative
22tc	F	63	Carotid	Somatic	*-*	Chr 11 −	Weak diffuse
56tc	F	50	Carotid	Germline	-	Chr 11 −	Weak diffuse
67tc	F	55	Carotid	Germline	-	Chr 11 −	Positive
68tv	F	68	Vagal	Germline	-	Chr 11 −	Weak diffuse
87tc	F	41	Carotid	Germline	-	N/A	Positive
100tc	F	35	Carotid	Germline	*NF1*—NM_000267: c.C5450G, p.S1817C (chr17:29654761, rs368654378) *	Chr 11 −	Weak diffuse
120tv	F	56	Vagal	Germline	-	Chr 11 −	Positive
135tc	F	50	Carotid	N/A	-	Chr 11 +	Positive
143tc	F	38	Carotid	Germline	-	Chr 11 −	Heterogeneous (+/− regions)
161tc	M	38	Carotid	Germline	-	N/A	Positive

* Germline variant; F, female; M, male; N/A, data unavailable.

## Data Availability

All data generated or analyzed in this study are included in the article.

## Supplementary Materials

The supporting information can be downloaded at: https://www.mdpi.com/article/10.3390/ijms24010628/s1.
