# Supplementary material for: The SDHD:p.H102R Variant Is Frequent in Russian Patients with Head and Neck Paragangliomas and Associated with Loss of 11p15.5 Region and Hypermethylation of H19-DMR
Source: Int J Mol Sci. 2022 Dec 30;24(1):628. doi: 10.3390/ijms24010628 (PMC9820527; doi:10.3390/ijms24010628)
Supplement: Supplementary file 1 [file ijms-24-00628-s001.zip › Table S1. Sequences of primers for microsatellite analysis..pdf]

Table S1. Sequences of primers for microsatellite analysis.

Microsatellite marker	Sequence (5'-3')
D11S1339	F: ATGGCCTTGGAAAAATATC
	R: GGGTGTAACCAGTTCTTCAG
D11S1347	F: CAGCCTGGGCAATAGT
	R: GCAGCAACAACAACAATAA
D11S927	F: AGTGAGCCGAGTTCGC
	R: ACCAAAAGCCTGGAATG
D11S5030	F: CTAAAGGATTGAGTCATGCCCTT
	R: GACAAGAGTGAGACCCTGTC
D11S1984	F: GGGTGACAGAGCAAAATTCT
	R: ACACCTGGATCTTGGACTCA
D11S4046	F: ACTCCAGCCTGGGAAAC
	R: TGATAGACACACCCATTGC
D11S4088	F: GGGCAGAGGCAGTGGAG
	R: GCATGTTTCGGGGGTG
D11S1318	F: CCCGTATGGCAACAGG
	R: TGTGCATGTNCATGAGTG
D11S901	F: CCCACATAGATTACTGGCCTC
	R: TCCTACATTAGCAGTTGGCA
D11S1313	F: CTAAGCATGANGCCAAGTTA
	R: AGTTTGACATTAGGGAATTTTGA
D11S907	F: GCTTATTGTCCATACCCAAA
	R: AAAGNACCTTAATTTCAGGC
D1S552	F: TTCATGCAGCATCATCCC
	R: TGTGGGCAGGTGTAAAGAGT

F, forward; R, reverse.
