# Supplementary material for: The SDHD:p.H102R Variant Is Frequent in Russian Patients with Head and Neck Paragangliomas and Associated with Loss of 11p15.5 Region and Hypermethylation of H19-DMR
Source: Int J Mol Sci. 2022 Dec 30;24(1):628. doi: 10.3390/ijms24010628 (PMC9820527; doi:10.3390/ijms24010628)
Supplement: Supplementary file 1 [file ijms-24-00628-s001.zip › Table S2. Sequences of converted DNA specific primers for differentially methylated regions (DMRs) on chromosome 11..pdf]

Table S2. Sequences of converted DNA specific primers for differentially methylated regions (DMRs) on chromosome 11.

DMRs	Sequence (5'-3')
H19-DMR	F: tcgtcggcagcgtcagatgtgtataagagacagGGTTATTTTTTTGTTTGAAGAT
	R: gtctcgtgggctcggagatgtgtataagagacagAATCTCCACTCCACTCCCAA
KvDMR	F: tcgtcggcagcgtcagatgtgtataagagacagTTGGTAGGATTTTGTTGAGGAGT
	R: gtctcgtgggctcggagatgtgtataagagacagCCCAATCAACAAATAAAAAAC

F, forward; R, reverse. Small letters notate sequences added for adapter annealing.
